# Editorial: Echocardiography in cardiovascular medicine

**DOI:** 10.3389/fcvm.2024.1427059

**Published:** 2024-05-21

**Authors:** Sanjeev Bhattacharyya, Francesca Innocenti

**Affiliations:** ^1^ Echocardiography Laboratory, St Bartholomew’s Hospital, London, United Kingdom; ^2^ Echocardiography Laboratory, Cleveland Clinic London, London, United Kingdom; ^3^ High-Dependency Unit, Department of Clinical and Experimental Medicine, Careggi University Hospital, Florence, Italy

**Keywords:** echocardiography, contrast, strain, artificial intelligence, three-dimensional

**Editorial on the Research Topic** Echocardiography in cardiovascular medicine

Echocardiography is one of the most versatile, non-invasive cardiac imaging modalities that facilitates accurate diagnosis, risk stratification, and guidance of therapy in every cardiovascular disease. The modality has evolved over several decades and encompasses a range of different techniques including deformation, contrast, and three-dimensional imaging as well as stress echocardiography and transoesophageal echocardiography. In this research topic, manuscripts have demonstrated both the breadth and depth of echocardiography in advancing our understanding of cardiovascular disease.

Identification of cardiac masses and distinguishing between non-malignant masses (thrombi, pseudo-tumours) and benign or malignant tumours is important. The diagnosis relies on a combination of clinical history, location of mass, and characteristics including mobility, morphology, whether the mass crossed tissues planes and the presence of pericardial effusion. Other modalities including positron emission tomography and cardiac magnetic resonance often provide complementary information. In certain cases, invasive biopsy is required. Li et al. show how using contrast enhanced echocardiography assessed using a quantitative approach (the ratio of peak contrast intensity of the mass compared to adjacent myocardium) can help differentiate between avascular thrombus (ratio close to zero) and malignant cardiac tumours (ratio > 1). Although the evidence base for contrast is strong, contrast is underutilised (1). Incorporation of contrast into routine echocardiographic practice for characterisation of cardiac masses should be encouraged as it can be performed rapidly and may result in less downstream testing if the result is conclusive.

Another area, which is gaining an increasing space in echocardiographic evaluation, is the employment of parameters of myocardial contractility. Conventional echocardiographic parameters, such as left ventricular ejection fraction (LVEF), maintain a definite diagnostic and prognostic value. They provide information on chamber performance, however they are highly dependent on pre- and afterload. Therefore, in the presence of diseases, like sepsis, where the loading conditions are altered, LVEF may be falsely normal, while myocardial contractility is depressed (Bagate et al., Nhat et al.). From this point of view, strain echocardiography appears to be a promising tool, as it requires a limited set of images evaluated during a standard echocardiographic assessment. It provides incremental diagnostic and prognostic roles, especially in the presence of a normal LVEF, where a subtle impairment of myocardial function may evolve and influence the outcome. In this situation, an early diagnosis could help identifying patients at risk of unfavourable evolution (Aboukhoudir et al., Lu et al., Schellenberg et al.). The analysis of the strain rate was applied on LV as well as right ventricle (RV) and left atrium (Mao et al.) by different authors who participated in the Research Topic. The analysis of RV systolic function is limited in conventional echocardiography, but its contribution to the prognostic assessment, in the presence of ischemic or valvular disease, is relevant. Winkler et al. showed that in patients undergoing transcatheter aortic valve implantation, a depressed RV global longitudinal strain was independently associated with an increased mortality. The evaluation of RV by strain echocardiography is actually confined to the research area, but we hope that in a near future, it could become widely available, as already happens for the LV. Nemes et al., showed three-dimensional speckle tracking can be used to map the tricuspid annulus and quantify right atrial function. The insights may help us understand the mechanisms of tricuspid valve disease.

Apical hypertrophic cardiomyopathy (HCM) characterised by hypertrophy localised to the left ventricular apex has a very variable clinical presentation. Anand et al. examined the prevalence and risk markers of pulmonary hypertension (estimated pulmonary artery systolic pressure > 36 mmHg) in a cohort of 542 patients with apical HCM at Mayo Clinic. They identified pulmonary hypertension was present in 34% of the cohort. Risk factors associated with pulmonary hypertension were female sex, atrial fibrillation, congestive heart failure and elevated filling pressure on echocardiography. The presence of pulmonary hypertension was associated with increased mortality. This knowledge may help better risk stratify patients with apical HCM. This will be important if therapeutic agents in apical HCM are identified in the future.

Artificial Intelligence (AI) is increasingly having an impact in all aspects of echocardiography from acquisition, detection of views, auto-measurement and quantification and more recently diagnosis of pathology. View classification is one of the first steps required for analysis of images. Most works showing the ability of convolutional neural networks to classify views has focussed on non-contrast images. Contrast enhanced echocardiography is often used in clinical practice to improve accuracy of left ventricular ejection fraction quantification. Zhu et al. showed a convolutional neural network can accurately classify both contrast-enhanced and non-contrast views. During the first wave of the COVID pandemic, emergency limited echocardiographic protocols were created to reduced exposure to COVID patients. Pellikka et al. examined the feasibility of incorporating automated AI analysis into these studies. In clinical reports, left ventricular ejection fraction (LVEF) was most commonly visually estimated (39%) and not quoted in 5%. Application of the AI with automated analysis of ejection fraction and longitudinal strain was feasible in 87% patients. In addition to improving the proportion of LVEF quantified, the AI quantified parameters were associated with outcomes. This study highlights how AI automated analysis can be used as a tool to improve quantification in echocardiography. Furthermore, Cheng et al. used a deep learning method to identify and grade the size pericardial effusion. These studies show the incremental progress being made in the field to improve workflow and quantification of pathology.

Echocardiography is the most widely used cardiovascular imaging modality. The topics has shown how echocardiography can be utilised in a range of different ways from a focussed study to a more comprehensive evaluation including strain, three-dimensional echocardiography or the employment of contrast medium. Appropriate training in conjunction with standard protocols for image acquisition and image processing are essential. However, artificial intelligence can be used as a tool to increase reproducibility and accuracy.
